# Probing the Molecular Dynamics of Aqueous Binary Solutions with THz Time-Domain Ellipsometry

**DOI:** 10.3390/s23042292

**Published:** 2023-02-18

**Authors:** Zahra Mazaheri, Gian Paolo Papari, Antonello Andreone

**Affiliations:** 1 Department of Physics “E. Pancini”, University of Naples Federico II, 80126 Naples, Italy; 2 Naples Unit, National Institute for Nuclear Physics, 80126 Naples, Italy

**Keywords:** THz sensing, ellipsometry, water solutions, hydrogen bonding

## Abstract

Using a customized time-domain ellipsometer operating in the THz range, the molecular dynamics of a liquid binary solution based on water and isopropyl alcohol (2-propanol) is investigated. The setup is capable of detecting small changes in the optical properties of the mixture within a single measurement. The complex dielectric response of samples with different concentrations is studied through the direct measurement of the ellipsometric parameters. The results are described using an effective Debye model, from which the relaxation parameters associated with different activation energies can be consistently extracted. Significant deviations between experimental data and the theoretical expectations at an intermediate volume percentage of 2-propanol in water are observed and interpreted as produced by competing effects: the creation/destruction of hydrogen bonding on the one hand, and the presence of cluster/aggregation between water and alcohol molecules on the other.

## 1. Introduction

Recently, there has been an increasing interest in the observation and study of the dielectric response of water and more specifically aqueous solutions in the terahertz (THz) region [1,2]. This is primarily due to two different reasons: (i) The molecular dynamics of water and its solutions with various substances at frequencies larger than microwaves are still vastly unexplored. THz time-domain spectroscopy (TDS) allows access to fast (ps and sub-ps) processes occurring in water mixtures; (ii) there is a need for the determination of the optical constants of liquids in this frequency range for many different biological, chemical, and engineering applications such as biosensing [3], solution chemistry [4], aquametry and aquaphotomics [5,6] agri-food [7,8], energy [9], protein hydrolysis [10], and many others.

Water-based mixtures, even though at first glance might be considered straightforward systems, have complex behavior in many aspects, which emerges in anomalous thermodynamic and transport properties [11,12,13]. The structures and function of macromolecules, alcohols, and water, while interacting with the environment, are highly affected by the presence of hydroxyl (OH) groups [14]. Alcohol molecules are amphiphilic in nature and induce opposite effects in water: In hydrophilic interactions, they favorably interact with water, forming hydrogen bonds (HBs), whereas through hydrophobic interactions, they tend to self-aggregate and disrupt the water structure via hydrophobic hydration. In mixtures of water and alcohol, the competition between hydrophobic and hydrophilic interactions along the alcohol chain length actually plays the main role, and over the years, it has been the subject of many numerical and computational studies [15] and investigated with different experimental techniques such as neutron diffraction [16], NMR [17], Raman, infrared spectroscopy [18,19], and THz-TDS [13]. It has been concluded that most results cannot be simply explained evoking the so-called “iceberg” model [20] and that the presence of hydrophobic moieties in the alcohol is responsible for the unusual mobility of water [21].

Investigating aqueous binary solutions using THz spectroscopy techniques allows probing the rotational and vibrational dynamics of molecules, as well as the translational motions caused by intermolecular interactions, especially HB formation [22,23]. As a matter of fact, THz spectroscopy is emerging as a label-free, nonionizing, innovative detection technique, potentially capable to shed light on the hydration dynamics of many biomolecules. However, water has a relatively high absorption coefficient in the spectral region under consideration, with a penetration depth of around 100 μm [24].

Measuring aqueous mixtures with a standard time-domain spectrometer, configured in transmission or reflection mode, is indeed challenging. The main hurdle in transmission measurements is the large losses, drastically reducing the signal-to-noise ratio (SNR) and consequently the analysis sensitivity. Moreover, the necessity of a thin (100 μm or less) cell to house the mixture implies (i) the risk that even a small thickness nonuniformity in the walls or air micro-bubbles in the container might produce spatial dependent measurements, and (ii) the presence of Fabry–Perot (FP) secondary oscillations in the time spectrum, which might disturb the nearby main signal. Besides that, material parameter extraction involves the careful comparison of the time-dependent electric field passing through the sample with respect to a reference signal (usually transmitted across an empty sample holder). As well, commonly used reflection or attenuated total reflection (ATR) measurements fail in terms of accuracy since in such schemes, the precision in sample-to-reference relative positioning is very critical [25]. In recent years, a number of time-domain measurements have been performed in the low THz band in both transmission configuration [2,26,27] and reflection or ATR configuration [25,28,29], aimed at revealing the low-frequency dynamics and mesoscopic structuring in water and aqueous solutions. In all these attempts, the key factor for the extraction of reliable and meaningful information on the dielectric properties of the liquids under test has been a judicious choice of the material and geometry of the cell and a careful post-measurement analysis in order to include (or dispose of) the FP multiple reflections between the cell window and sample interface.

Recently, THz time-domain spectroscopic ellipsometry (THz-TDSE) has been considered a novel and powerful technique in studying liquids such as polar fluids [30,31,32]. While retaining the advantage of (specular or variable angle) reflection methods in removing inherent limitations given by material loss, THz-TDSE presents the additional benefit of being a self-reference technique, thus improving the measurement accuracy since it is free of errors due to possible sample misplacement. Moreover, with respect to optical ellipsometry, THz-TDSE relies on the simultaneous acquisition of both amplitude and phase of the electric field of a short (ps) pulse, providing a coherent spectroscopic analysis that enables researchers to obtain meaningful information on losses resulting from both localized (refractive index) and delocalized (extinction coefficient) charge. In this way, one can easily probe the molecular dynamics of liquid solutions within a single measurement.

In this work, using a customized ellipsometer operating in the THz range [33], a set of measurements are presented that were carried out on mixtures based on water and isopropyl alcohol (2-propanol) with different volume concentrations. The aim of the study is to show that the TDSE technique can be effectively used to describe the mechanisms governing the molecular dynamics in binary solutions consisting of polar liquids. After a brief introduction to the TDSE setup, the relations between the measured ellipsometric parameters and the dielectric response of the material under test are described. Then, the experimental results are shown, and a discussion is presented in terms of an effective Debye model for interacting liquids. Finally, data are analyzed in order to extract direct information on the different activation energies associated with inter-molecular interactions, and the results are discussed in terms of the competition among hydrogen bonding, clusterization, and aggregation effects in the water–alcohol mixtures.

## 2. Materials and Methods

**Figure 1:**
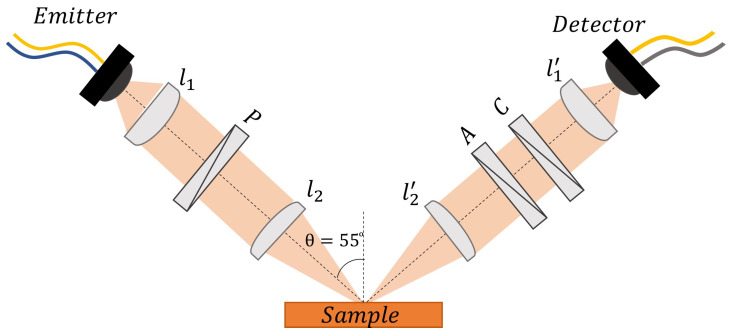
The horizontal THz-TDSE setup used to carry out the measurements of the binary liquid solutions.

Figure 1 illustrates the optical setup used to carry out the measurements when the sample was placed in a horizontal position. The beam angle of incidence θ varied between 20° and 80°. Two fiber-coupled photoconductive antennas were used to generate (emitter) and coherently probe (detector) the THz electric field. The optical setup included two pairs of polymethylpentene (TPX) lenses having different *f*-numbers (1.31 in the case of $l_{1},l_{1}^{\prime }$, 2.62 in the case of $l_{2},l_{2}^{\prime }$) to collimate and focus the beam. The control of the polarization of the THz signal ess realized using three free-standing wire-grid polarizers (WGP), with broadband transmission and extinction ratio ranging between 20 and 50 dB. The WGPs are mounted on motorized, computer-controlled rotational stages ensuring a very precise orientation (within $\pm 0.1^{\circ }$) to the desired azimuthal angle. Placing the emitter at $45^{\circ }$ with respect to the plane of incidence and the first polarizer “P” oriented at $\phi_{P}=-45^{\circ }$ guaranteed that a perfectly linear polarized THz signal with equal s- and p-polarized components would reach the sample surface. The second polarizer “A” was placed in the optical path immediately after the beam was reflected back, and it served as a selector between p- or s-polarized components, setting $\phi_{A}=0^{\circ }$ or $\phi_{A}=90^{\circ }$, respectively. An additional polarizer “C”, with finely variable rotation, was used to compensate for any possible error caused by misorientation in the optical setup or nonideal features of the beam, in addition to providing the receiving antenna, which was oriented at $45^{\circ }$, with the same sensitivity of detection in and out of the plane of incidence for the two polarization components.

Under this configuration, the system is able to record the time profile of both s- and p-polarized components of the THz electric field reflected from the sample with high accuracy, and subsequently, the corresponding frequency spectra are evaluated via fast Fourier transform (FFT) analysis. In this way, the ellipsometric parameters Ψ and Δ, expressing, respectively, the amplitude ratio and the phase difference of the s- and p-polarized reflected components as a function of frequency, can be directly acquired [31]. Ψ and Δ are in turn linked to the complex optical response $\tilde{N}\left(\omega \right)=n\left(\omega \right)-ik\left(\omega \right)$ of the (assumed nonmagnetic) sample under investigation via the following equations [34]:

(1) $$n^{2}-k^{2}=\sin^{2}\theta \left[1+\frac{\tan^{2}\theta \left(\cos^{2}2\Psi -\sin^{2}2\Psi \sin^{2}\Delta \right)}{{\left(1+\sin2\Psi \cos\Delta \right)}^{2}}\right]$$

(2) $$2nk=\sin^{2}\theta \frac{\tan^{2}\theta \sin4\Psi \sin\Delta }{{\left(1+\sin2\Psi \cos\Delta \right)}^{2}}.$$

where *n* and *k* represent the refractive index and extinction coefficient, respectively. To improve the accuracy in the evaluation of the optical response [31], the beam’s incident angle θ was set at 55°, in close proximity to the pseudo–Brewster angle $\theta_{B^{\prime }}$ of isopropyl alcohol (≈58° in the frequency range under investigation).

To probe the liquid binary solutions based on water and isopropyl alcohol with different volume concentrations Vc, the optical properties of pure isopropyl alcohol (ROMIL Ltd., Cambridge, UK, purity higher than 99.7%) and deionized water (Milli-Q grade) were first extracted by directly measuring the ellipsometric parameters (Ψ; Δ) and using Equations (1) and (2). After this first analysis, to prepare the binary mixtures with different volume concentrations (see Table 1), isopropyl alcohol was gradually added to 20 mL of deionized water using a standard graduated syringe with an accuracy of 0.05 mL. Usually, the properties of binary mixtures are studied by varying the molar fractions of the constituents. However, following previous reports [35,36], when dealing with the optical response of a homogeneous aqueous solution, the volume fraction represents a more appropriate measure of concentration than a molar fraction. The main reason is that the effective path length used for probing the dielectric behavior is determined for each species by the corresponding volume fraction.

## 3. Results and Discussion

In this study, we focused on the water-rich region (low alcohol concentration) since the TDSE setup covers a relatively narrow spectral range and therefore, as shown in subsequent sections, can temporally probe only the intermediate relaxation events occurring in the mixture, lying in between the fast (ps) and slow (hundreds of ps) molecular vibrations of water and alcohol, respectively.

The optical properties of each sample were measured under the same conditions as those for the pure constituents. The liquid solutions were put in Petri dishes with 14 mm thickness and 88 mm diameter. The dimensions of the container ensured both a sufficiently large surface area for the impinging signal and a relatively considerable depth in order to provide enough absorption and exclude the interference produced by the secondary signal reflected from the lower liquid–dish interface. The presence of back reflections in fact might affect the results of ellipsometric measurements. All measurements were carried out in a closed environment at a stabilized temperature of 23 ± 1 °C. Each time-domain signal reflected from the sample was averaged over 1000 times, which usually takes less than a minute.

In pure liquids, TDSE data were also confirmed through independent measurements performed on the same samples in the transmission configuration mode, where a cuvette with a 100 μm pool and quartz window plates (Hellma^®^ Analytics) was used as the sample holder.

**Figure 2:**
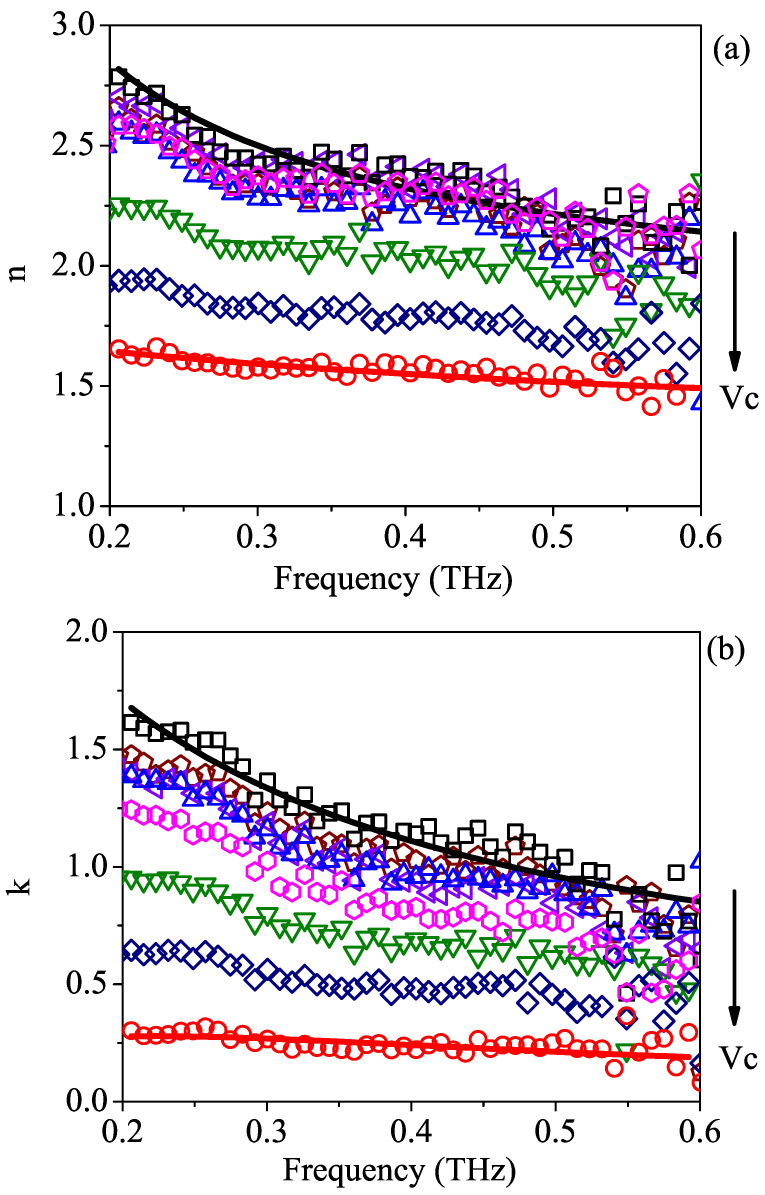
The frequency dependence of (**a**) refractive index *n* and (**b**) absorption coefficient *k* obtained from TDSE measurements for aqueous binary solutions with different volume concentrations Vc of isopropyl alcohol. Dashed curves represent the results of a fitting procedure performed on pure water (red continuous line) and pure 2-propanol (black continuous line) using the generalized Debye relaxation model. The volume concentration increases from 0% (water, □) to 100% (2-propanol, ∘) according to the values listed in Table 1. The arrow indicates the increasing trend for Vc.

Figure 2a,b illustrate the optical properties of the samples listed in Table 1. The solid points reflect the results that were experimentally achieved using the THz-TDSE technique, whereas the dashed lines correspond to the results of the fit procedure applied to pure water and isopropyl alcohol data using the Debye relaxation model, which will be discussed in the following section. As expected, by adding isopropyl alcohol to water, the refractive index and the absorption coefficient of the polar mixture gradually diminished. The arrows show the decreasing trend in both parameters with the increase in volume concentration of isopropyl alcohol in water. At the very high end of the investigated frequency spectrum, the data show an increased scattered behavior, which is attributed to the low signal-to-noise ratio produced by extra losses and dispersion in the laser beam [33]. It is also worth noting that the setup allowed us to discern a very small amount of isopropyl alcohol (as low as 1%) in a water solution in a simple way and within a single measurement.

Using a quasi-static approach, the dielectric function of binary solutions can be predicted using mixing models such as Maxwell–Garnett, Bruggeman, or Kirkwood [37]. For instance, the standard Maxwell–Garnett (MG) model describes the mixture as spherical inclusions (guest phase) embedded in a background medium (host phase). Under these assumptions, the complex dielectric response $\tilde{\epsilon }\left(\omega \right)=\epsilon {}^{\prime }\left(\omega \right)+i\epsilon {}^{\prime \prime }\left(\omega \right)$ of such a medium can be effectively expressed in terms of the effective volume concentration [37] as follows:

(3) $${\tilde{\epsilon }}_{eff}\left(\omega \right)={\tilde{\epsilon }}_{e}\left(\omega \right)+3Vc{\tilde{\epsilon }}_{e}\left(\omega \right)\frac{{\tilde{\epsilon }}_{i}\left(\omega \right)-{\tilde{\epsilon }}_{e}\left(\omega \right)}{{\tilde{\epsilon }}_{i}\left(\omega \right)+2{\tilde{\epsilon }}_{e}\left(\omega \right)-Vc\left({\tilde{\epsilon }}_{i}\left(\omega \right)-{\tilde{\epsilon }}_{e}\left(\omega \right)\right)}$$

where ${\tilde{\epsilon }}_{i}$ and ${\tilde{\epsilon }}_{e}$ represent the complex dielectric function of the inclusion and its environment, respectively. It is worth emphasizing that the fundamental physical assumption in these models is that the components in the mixture do not interact.

In the case of liquids, the Debye theory has been proven over the years to successfully predict the response of solutions containing permanent electric dipole moments. The most significant parameter of the model is the relaxation time (τ), which describes the response of the polar molecules reorienting themselves in the medium once the external field is switched off [37]. In the case of pure polar liquids such as water or isopropyl alcohol, the Debye model can be effectively used to study the dielectric properties of the sample. The relaxation process related to simple and independent rotational events is relatively fast in water (described by $\tau_{1,w}$) and much slower in alcohol (described by $\tau_{1,iso}$), where it has been clearly observed in the microwave region [12]. In the THz region, fast relaxation processes associated with the breaking and formation of hydrogen bonds take place and need to be considered. Alcohols present hydroxyl groups both at the end and within a chain, with the former less likely to produce a switching event between adjacent molecules. Liquid 2-propanol dynamics is therefore governed by three relaxation events, a slow mechanism due to molecule rotational movement, an intermediate process, and a faster process corresponding to the switching of H-bonds at the end and within each chain, described by $\tau_{2,iso}$ and $\tau_{3,iso}$, respectively. Water, due to the wide surrounding HB network, favors an easy switching of H-bonds between a molecule and its neighbors, and has therefore only a second, faster relaxation event. As it will be better shown later in this section, for clarity, this event is described by the term $\tau_{3,w}$.

When multiple relaxation events are present, in order to accurately describe the complex dielectric response of the liquid, the generalized Debye Equation [38] is used as follows:

(4) $$\tilde{\epsilon }\left(\omega \right)=\epsilon_{\infty }+\sum_{i=1}^{N}\frac{\epsilon_{i}-\epsilon_{i+1}}{1+i\omega \tau_{i}}$$

This formula shows that in a liquid *N*, distinct processes contribute each with a relaxation time $\tau_{i}$ and dielectric strength $\epsilon_{i}-\epsilon_{i+1}$, where $\epsilon_{1}=\epsilon_{s}$ is the static dielectric constant, and $\epsilon_{N+1}=\epsilon_{\infty }$ is the residual independent dipoles permittivity at very high frequencies. In the case of water and isopropyl alcohol, N=2 and N=3, respectively.

Using Equation (4) and a nonlinear regression algorithm, the experimental data of pure water and 2-propanol versus frequency were fitted, yielding the relaxation and dielectric parameters listed in Table 2. The corresponding curves are represented by the dashed lines displayed in Figure 2a,b, for the refractive index and absorption coefficient, respectively. Even if the Debye model has the potential to give useful information regarding the molecular dynamics in liquid systems, the observed THz spectral range is too small to provide a reliable quantitative analysis on both relaxation strengths and times. For this reason, in the fit procedure, all dielectric strength values were kept fixed to the corresponding values in the literature [12], whereas the infinite frequency permittivity was set at a value extrapolated from the experimental data. Relaxation times are only given as free parameters. For isopropyl alcohol, since the liquid dynamics were probed in a relatively high-frequency region, the slowest relaxation time $\tau_{1,iso}$ was also taken from [12]. The results confirm the scenario described above in terms of slow and fast relaxation processes, yielding a value for $\tau_{1,w}$ in water that is orders of magnitude larger than $\tau_{3,w}$, while in isopropyl alcohol, $\tau_{2,iso}>\tau_{3,iso}$, with values ranging in the ps range. This last finding is consistent with the studies carried out in [13].

In previous reports, the simplest way to extend the Debye model to a liquid solution was to treat it as an “ideal” mixture, where the dielectric properties of the resulting medium are described using a simple additive rule starting from the characteristics of neat liquids [13,36,39]. This approach is based on the ansatz that the two (or more) liquids composing the mixture are not interacting and is therefore perfectly equivalent to the description based on the MG formula given in Equation (3).

However, in the case of aqueous mixtures, the coupling of intra- and inter-molecular vibrations of water may potentially delocalize the vibrational modes of the solute [40], eventually altering the dielectric response of the solution. In the THz region, molecular interactions such as intersecting HB networks, the packing density of foreign molecules, or hydration dynamics cannot be neglected. Therefore, the dielectric response of water-based mixtures, and more generally polar liquids, cannot be predicted by the abovementioned models.

The first analysis of an *effective* Debye relaxation model for mixed solutions was conducted by Lou et al. [41], grounding their study, however, on the slow relaxation terms only. Recently, Zhou and Arbab [27] described the dynamics of binary water–alcohol solutions introducing a three-term effective Debye relaxation model to predict the complex dielectric function $\tilde{\epsilon }\left(\omega \right)$ in the THz region. To take into account the presence of two different polar liquids in the mixture, the parameters in Equation (4) are replaced by effective values, calculated using empirical rules (the symmetric Bruggeman Equation [37] for the dielectric strengths) and physically sound assumptions, with each contribution from the solvent and the solute weighted in terms of volume concentration Vc. In the case of the effective relaxation times for an ideally mixed solution, it is expressed as follows [27]:

(5) $$ln\left(\tau_{i,eff}\right)=Vc\;ln\left(\tau_{i,iso}\right)+\left(1-Vc\right)\;ln\left(\tau_{i,w}\right))$$

where *i* = 1 or 3, respectively, depending if the slow or fast dynamical events are cooperatively involved. In this effective model, therefore, water and alcohol molecules as a whole are virtually treated as a completely mixed inter-species HB network.

Since water has only two relaxation events, the intermediate effective term [$\left(\epsilon_{2,eff}-\epsilon_{3,eff}\right)/\left(1+i\omega \tau_{2,eff}\right)$] in Equation (4) is provided setting $\epsilon_{2,eff}=\epsilon_{2,iso}$ and $\tau_{2,eff}=\tau_{2,iso}/Vc$ [27]. The latter relation accounts for the fact that due to the decrease in the volume concentration, fewer and fewer 2-propanol molecules are available for HB switching at the end of the chains, so the corresponding effective intermediate relaxation time increases.

Then, using the effective Debye model and the values of the relaxation parameters listed in Table 2 for the pure liquids, the dielectric response of the binary solutions varying Vc can be predicted at every frequency using the following equation:

(6) $$\hat{\epsilon }=n^{2}-k^{2}+i2nk$$

**Figure 3:**
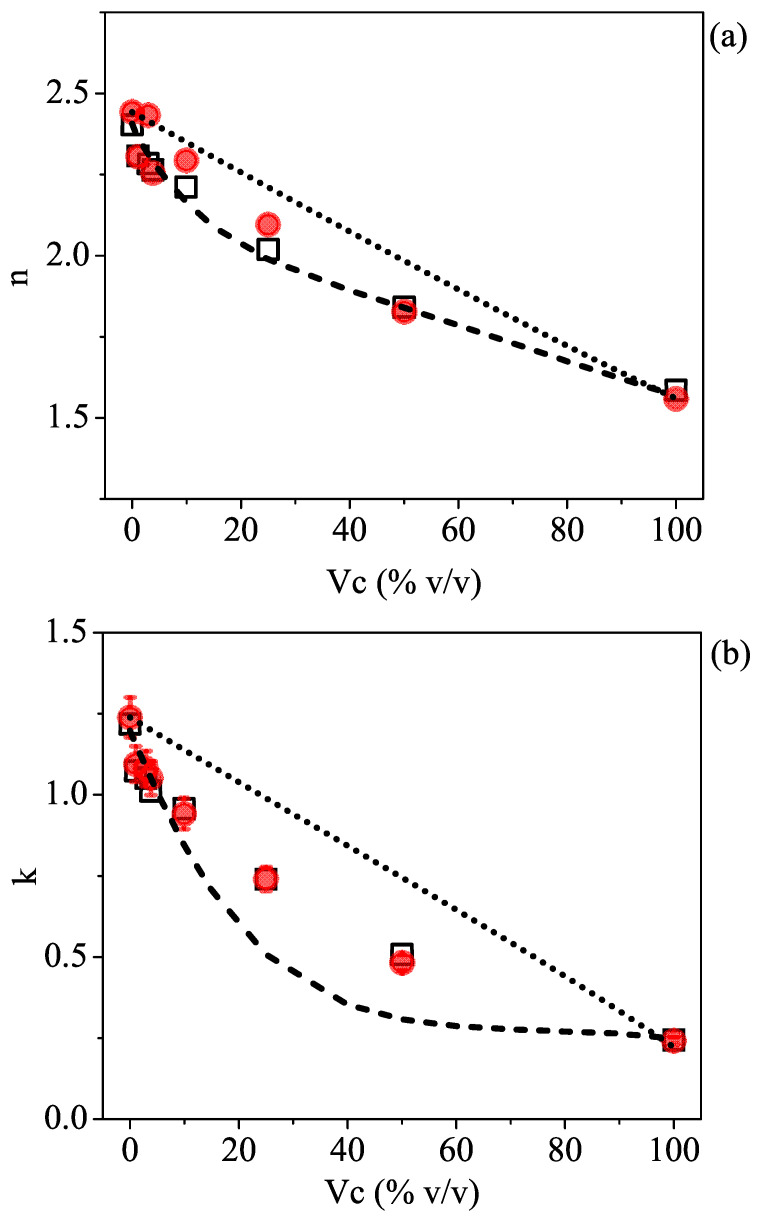
(**a**) *n* and (**b**) *k* plot vs. Vc showing the comparison between experimental (red circles) and theoretical values expected from the Maxwell–Garnet model (dotted line) for noninteracting mixtures and from the effective Debye relaxation model (dashed line) using the dielectric parameters of the pure constituent liquids, water, and isopropyl alcohol. In the graph, the results (open black squares) directly obtained by fitting (see text) the ellipsometric data at 0.35 THz with the effective Debye relaxation model are also shown.

To check the validity of the model, the last step was to compare the estimated values for *n* and *k* with the results obtained from ellipsometric measurements. Figure 3a,b display the TDSE experimental values (full red circles) for the refractive index and the absorption coefficient, respectively, taken at a given frequency (0.35 THz) as a function of the 2-propanol volume concentration, together with the theoretical expectations from the effective Debye relaxation model (dashed curve). The linear dependence describing the Maxwell–Garnett mixing model for a noninteracting solution and expressed by Equation (3) is reported as a dotted line.

Data comparison shows three distinct features: (i) The effective Debye model can describe the decrease in the refractive index and the absorption coefficient of the polar mixture as a result of gradually adding isopropyl alcohol in the water much better than a noninteracting molecule model; (ii) at very low-volume concentrations (below 20%), data closely follow the Debye model, but they start to depart from the expected behavior at larger Vc values; (iii) within the accuracy given by the measurement error in the ellipsometric parameters [31], the mismatch is far more evident in the *k* vs. Vc plot (Figure 3b).

All these findings are consistent with previously reported data for water/alcohol [13] and other binary mixtures [35] that the behavior of the mixture at intermediate volume concentrations of alcohol departs from what is predicted by an “ideal” (noninteracting) model. This mostly results in a decrease in the expected terahertz absorption, associated with a retarded rotational dynamics and an increase in the structuring of the extended HB networks between the host and guest molecules [36]. As a consequence, *k* is affected, whereas *n* is not as much.

In water solutions, the amphiphilic nature of monohydric alcohols, due to the presence of both hydrophilic and hydrophobic groups, might lead to both destructive and cooperative interactions. These two opposing effects combine together to modify the extensive HB network of water depending on the composition of the binary mixtures.

Nevertheless, the observed results show that neither the MG model for a noninteracting solution nor the effective Debye theory applied to an ideally mixed solution can explain the experimental data.

Indeed, the difference in absorption could also arise from a decrease in the amount of hydrogen bonding in the mixtures as well. In their neutron diffraction studies, Dixit et al. [16] found that it is the strength, not the number of hydrogen bonds, that changes the addition of alcohol to pure water. Contrary to the speculation that the water structure is either enhanced or destroyed in an alcohol solution, the local structure of water in a concentrated methanol–water solution studied in [16] was very similar to the one in pure water. The conclusion was that the anomalous thermodynamic properties of alcohol–water mixtures arise from incomplete mixing as a result of cluster formation as well as the self-aggregation of the alcohol [42,43], rather than from water restructuring.

According to various authors [35,36,44], the effect of H-bonding in aqueous isopropyl alcohol mixtures primarily appears to be in the slow relaxation time, as it confines the rotational movements of the molecules. To this aim, the experimental data observed for the solutions with different volume concentrations were directly fitted to the effective Debye model, setting $\tau_{1,eff}$ as a free parameter. The results are shown as open black squares in Figure 3. By only changing the effective relaxation time describing the slow molecular dynamics in the solution, a very good matching between the estimated and experimental data, both in *n* and *k* dependence, is observed.

**Figure 4:**
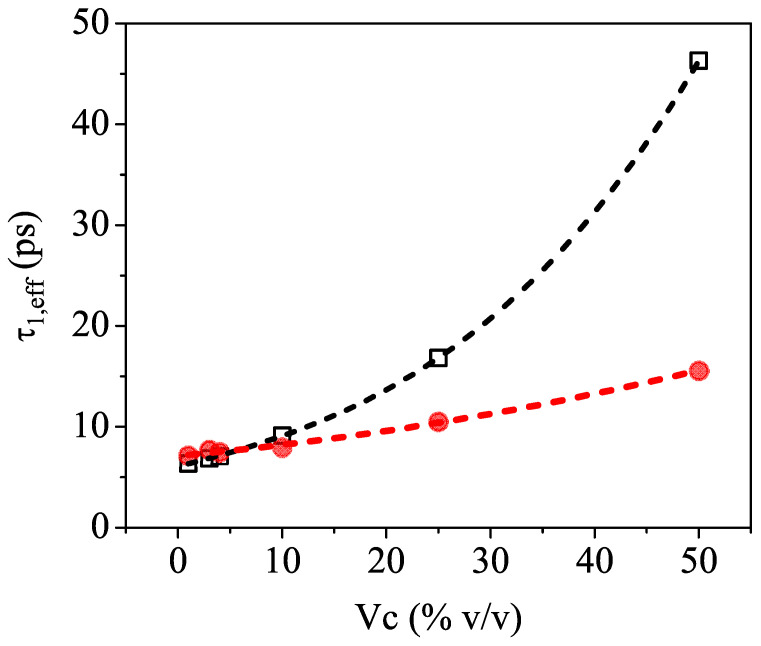
Comparison between the values of the effective relaxation time $\tau_{1,eff}$ calculated (black open squares) and directly extracted (red full circles) from the effective Debye model fit of the experimental values (*n* and *k* vs. frequency) as a function of the volume concentration Vc of the binary mixture. The dashed lines are used as guides for the eye only.

Figure 4 shows $\tau_{1,eff}$ as a function of Vc and clearly indicates that relaxation time values extracted from the fit (red full circles) are significantly smaller than the calculated ones (black open squares) based on the ansatz of an ideally mixed solution of pure water and pure isopropyl alcohol.

We believe that this behavior is an indirect indication of incomplete mixing, which leaves its signature in the THz frequency region. Indeed, previous reports [13,16,39] showed that water–alcohol mixtures are not homogeneous throughout the entire concentration range. There are in fact two competing effects governing the molecular dynamics in the aqueous solutions, which are produced by the amphiphilic nature of the 2-propanol molecules. The hydrophilic mechanism facilitates hydrogen bonding with water, whereas the hydrophobic interaction induces the self-aggregation of the molecules as well as cluster formation, thus disrupting the water structure [43]. It is, therefore, expected that the inhomogeneous mixing will affect the long-range collective H-bonding dynamics of water [44], and this is exactly what is observed in the slowing down of the relaxation events measured in the sub-THz frequency region. It is, however, important to emphasize that TDSE measurements cover only the high-frequency end of the dielectric relaxation spectrum, so they cannot clearly distinguish between multiple rotational movements or switching events. Therefore, the relaxation times $\tau_{eff,1}$ obtained from the fit using an effective Debye theory should be regarded as indicative values, useful only to compare and mark the difference with the numbers expected from the model.

## 4. Conclusions

In this work, the high-frequency (sub-THz) behavior of a binary solution consisting of Milli-Q water and isopropyl alcohol (2-propanol) with different volume concentrations Vc was measured using a TDSE technique. The customized setup we built is capable to detect small changes in the optical response of the sample and to probe its molecular dynamics within a single measurement. Firstly, the dielectric response of the neat liquids as the constituents of the mixture was analyzed using a generalized Debye relaxation model. Then, the optical properties of solutions upon increasing Vc were calculated using an effective Debye relaxation model. THz ellipsometric measurements indicate a strong deviation in the absorption coefficient of the alcohol–water binary mixtures from both an ideal (noninteracting) and a fully mixed solution, which to our understanding, is a manifestation of the competition among the creation and destruction of H-bonds, water–alcohol clusterization, and alcohol–alcohol aggregation. It must be emphasized that the Debye analysis is limited by the currently accessible spectral range probed by terahertz spectroscopy. Moreover, the development of accurate models to describe the relaxation processes in binary mixtures is not trivial, and there is a need for appropriate equations that accurately represent the relaxational/vibrational characteristics of each system. 

## Figures and Tables

**Table 1:** Water–isopropyl alcohol binary solutions with different volume concentrations.

Water Volume (mL)	Isopropyl Alcohol Volume (mL)	*Vc* (% *v*/*v*)
20	0	0
20	0.2	1
20	0.6	3
20	0.8	4
20	2.2	10
20	6.5	25
20	20	50
0	20	100

**Table 2:** Debye parameters of pure water and isopropyl alcohol.

Sample	$\epsilon_{\infty }$	$\epsilon_{s}{\;}^{a}$	$\epsilon_{2}{\;}^{a}$	$\epsilon_{3}{\;}^{a}$	$\tau_{1}$ (ps)	$\tau_{2}$ (ps)	$\tau_{3}$ (ps)
water	3.06	78.36	-	4.00	6.1 ± 0.5	-	0.20 ± 0.02
isopropyl alcohol	2.05	19.42	4.75	3.86	350${\;}^{a}$	5.5 ± 0.9	1.0 ± 0.4

${\;}^{a}$ Values taken from [12].

## Data Availability

The data presented in this study are available on request from the corresponding author.
